# Ultrasensitive colorimetric detection of circulating tumor DNA using hybridization chain reaction and the pivot of triplex DNA

**DOI:** 10.1038/srep44212

**Published:** 2017-03-09

**Authors:** Ruimin Li, Li Zou, Yanwei Luo, Manjun Zhang, Liansheng Ling

**Affiliations:** 1 School of Chemistry, Sun Yat-Sen University, Guangzhou 510275, P. R. China

## Abstract

This work presents an amplified colorimetric biosensor for circulating tumor DNA (ctDNA), which associates the hybridization chain reaction (HCR) amplification with G-Quadruplex DNAzymes activity through triplex DNA formation. In the presence of ctDNA, HCR occurs. The resulting HCR products are specially recognized by one sequence to include one GGG repeat and the other containing three GGG repeats, through the synergetic effect of triplex DNA and asymmetrically split G-Quadruplex forming. Such design takes advantage of the amplification property of HCR and the high peroxidase-like catalytic activity of asymmetrically split G-Quadruplex DNAzymes by means of triplex DNA formation, which produces color signals in the presence of ctDNA. Nevertheless, in the absence of ctDNA, no HCR happens. Thus, no triplex DNA and G-Quadruplex structure is formed, producing a negligible background. The colorimetric sensing platform is successfully applied in complex biological environments such as human blood plasma for ctDNA detection, with a detection limit corresponding to 0.1 pM. This study unambiguously uses triplex DNA forming as the pivot to integrate nucleic acid amplification and DNAzymes for producing a highly sensitive signal with low background.

Circulating tumor DNA (ctDNA), composed of small fragments of nucleic acid, has been demonstrated to be a promising biomarker for classification, diagnosis, and prognosis of cancers, which attracts much attention to develop techniques for monitoring ctDNA in blood^1^,^2^,^3^. Polymerase chain reaction (PCR) detection methodology represents a classical technology for quantifying ctDNA^4^, but it is costly and requires thermal cycling, complex, tedious steps and sophisticated instruments. Many isothermal nucleic acid amplification techniques, including rolling-circle amplification (RCA)^5^, strand-displacement amplification (SDA)^6^, helicase-dependent amplification (HAD)^7^ and hybridization chain reaction (HCR)^8^ have the potential to revolutionize detection and monitoring of ctDNA. Among them, HCR, an enzyme-free amplification technique, has superior strengths, such as, less hassle in operating conditions, cost effectiveness, sensitivity and simplicity^9^,^10^. Based on HCR amplification, a variety of targets have been successfully determinated, including nucleic acids, small molecules and proteins^11^,^12^,^13^.

For instance, Miao *et al*. proposed two strategies for miRNA detection based on hybridization chain reaction. One took advantage of the strategy that analyte triggered nanoparticle localization on a tetrahedral DNA modified followed by HCR amplification^14^, the other is based on HCR-mediated localized surface plasmon resonance (LSPR) variation of silver nanoparticles (AgNPs)^15^. Xie *et al*. developed an aptasensor assay for lipopolysaccharides based on HCR^16^. Zou *et al*. reported an universal platform for nucleic acids, small molecules and proteins, which integrated HCR with the assembly of gold nanoparticles (AuNPs)^17^. Although there are many advances about HCR in the design of biomarker assays, it still needs to develop new methods, which can efficiently amplify target and produce a highly sensitive signal with zero background.

Here, we proposed an amplified colorimetric strategy for highly selective and sensitive detection of the ctDNA, which employed the triplex forming oligonucleotides (TFOs) to specially recognize the dsDNA products of HCR and synergistically form the asymmetrically split G-Quadruplex structure. The resulting G-Quadruplex structure bound with hemin and formed the asymmetrically split G-quadruplex/hemin complex with high peroxidase-like activity, which catalyzed the oxidation-reduction reaction of 2,2′-azino-bis(3-ethylbenzothiozoline-6-sulfonic acid) (ABTS^2−^) and H_2_O_2_, and yielded the green radical anion (ABTS^·−^) and H_2_O. Consequently, a detectable color change was achieved by the naked eye or with a spectrophotometer. To the best of our knowledge, we have for the first time used the triple-helix DNA forming technique to combine the HCR products and the asymmetrically split guanine-rich DNA sequences, and achieved colorimetric and visual detection of the ctDNA sequence with highly sensitive signal and negligible background.

## Results and discussion

### Principle of the proposed method

**Figure 1:**
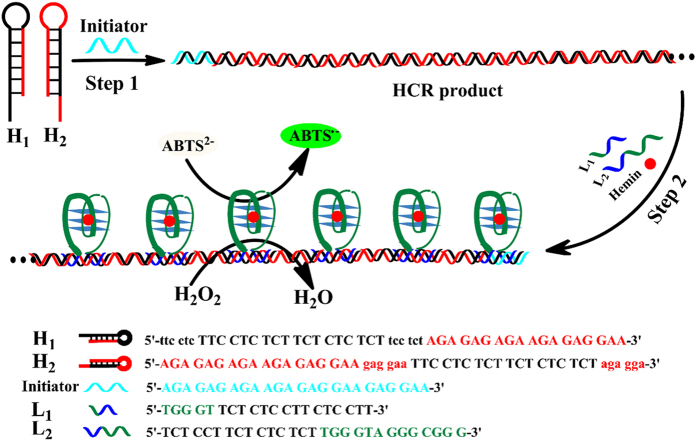
Schematic illustration of amplified colorimetric nucleic acid detection.

The principle of the proposed colorimetric sensing system is depicted in Fig. 1. It involves five oligonucleotides, H_1_, H_2_, Initiator, L_1_ and L_2_. H_1_ and H_2_ are designed to contain the hairpin structures and have 18-base stems, 6-base loops and 6-base sticky ends, which are stable and have no chance to hybridize with each other at room temperature. When the sequence Initiator is introduced in the system, the steady state of H_1_ and H_2_ is disturbed by the hybridization of Initiator and H_1_, and then triggering a chain reaction for alternating addition of these two hairpins, yielding a chain-like assembly of H_1_ and H_2_. The HCR product with a strict duplex formation has an iterative 30-base homopyrimidine·homopurine druplex DNA region, which is specifially recognized by a third homopyrimidine or homopurine single-strand DNA by Hoogsteen or reverse Hoogsteen hydrogen resulting in the structure of triplex DNA. The sequence L_1_ has two portions, a portion with 15-base homopyrimidine, which has the capability of sequence-specific recognition of the major groove of homopyrimidine·homopurine druplex DNA of HCR product, the other portion with the one-fourths of the G-quadruplex sequence. Similarly, L_2_ also has two functional parts, one part with 15-base homopyrimidine that can recognize homopyrimidine·homopurine druplex DNA of HCR product and form the triplex DNA, the other one to include the three-fourths of the G-quadruplex sequence^18^. Under certain conditions, the homopyrimidine parts in L_1_ and L_2_ specially recognize the major groove of homopyrimidine·homopurine dsDNA in the HCR product. Based on the simple rule of complementary Hoogsteen hydrogen bonding, the triplets, CG·C^+^ and TA·T, are obtained^19^. In other words, the triplex DNA is successfully formed by HCR products, L_1_ and L_2_. Meanwhile, the two asymmetrically split parts in L_1_ and L_2_ assemble to form a special G-quadruplex structure. The special G-quadruplex structure can bind with hemin and form the asymmetrically split G-quadruplex/hemin complex with high peroxidase-like activity, which can catalyze the reaction of ABTS^2−^ and H_2_O_2_. Thus, a detectable color change is achieved by the naked eye or with a spectrophotometer.

### Feasibility of the proposed method

**Figure 2:**
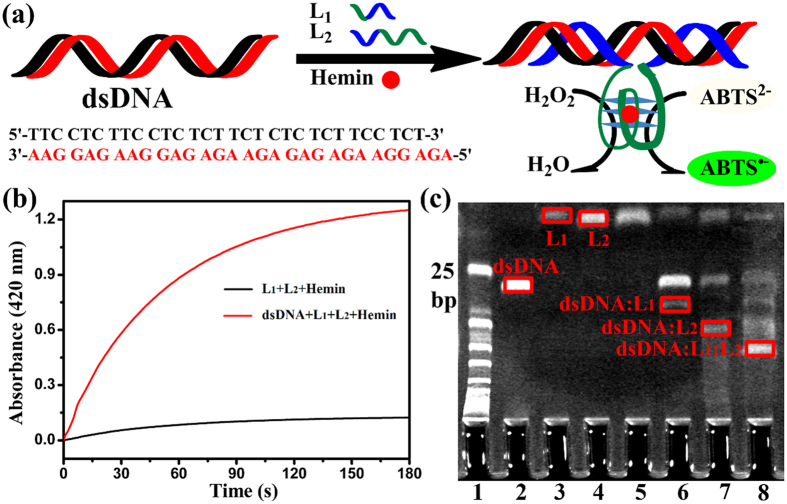
(**a**) Analysis of the iterative (dsDNA) in HCR products by L_1_ and L_2_ that produced the triplex DNA and asymmetrically split G-quadruplex configurations through the synergistic effect. (**b**) Comparison of time-dependent absorbance changes of ABTS^·−^ upon the absence (black) and presence (red) of dsDNA. (**c**) The effect of the triplex DNA forming by L_1_, L_2_ and the iterative (dsDNA) in HCR products. Lane 1, 25-bp ladder marker; lane 2, 0.5 μM dsDNA; lane 3, 1 μM L_1_; lane 4, 1 μM L_2_; lane 5, 1 μM L_1_ and 1 μM L_1_; lane 6, 0.5 μM dsDNA and 1 μM L_1_; lane 7, 0.5 μM dsDNA and 1 μM L_2_; lane 8, 0.5 μM dsDNA, 1 μM L_1_ and 1 μM L_2_.

To clearly demonstrate the triplex DNA forming by L_1_, L_2_ and HCR products, we chose the iterative 30-base homopyrimidine·homopurine druplex DNA region in HCR products as the target dsDNA, as shown in Fig. 2a. In the presence of dsDNA, L_1_ and L_2_ recognized the major groove of homopyrimidine·homopurine. Through the synergistic effect, the triplex DNA and asymmetrically split G-quadruplex configurations were formed by dsDNA, L_1_ and L_2_. The special G-quadruplex structure bound with hemin and became an active DNAzyme. As depicted in Fig. 2b, in the absence of dsDNA, the absorbance value of the oxygenation product ABTS^·−^ at 420 nm was about 0.1 at the 180 seconds time point. However, in the presence of dsDNA, the value was about 1.2. Therefore, it indicated that the two asymmetrically split G-rich parts of L_1_ and L_2_ assembled to form an active G-quadruplex structure in the presence of the homopyrimidine·homopurine sequences dsDNA. Furthermore, to investigate the configuration change of this process, we carried out the native polyacrylamide gel electrophoresis (PAGE) under different conditions, with dsDNA, L_1_ and L_2_ as the controls. As exhibited in Fig. 2c, in the absence of dsDNA, L_1_ and L_2_ had no connection with each other (lane 5). By the introduction of dsDNA, the resulting triplex DNA products, dsDNA: L_1_ (lane 6), dsDNA: L_2_ (lane 7), dsDNA: L_1_ : L_2_ (lane 8), were obtained. Since the homopyrimidine parts in L_1_ and L_2_ had the ability of sequence-specific recognition of the major groove of homopyrimidine·homopurine dsDNA. Thus, the rationale that introduced the asymmetrically split G-quadruplex/hemin DNAzymes as the catalyst of producing signals with the aid of forming triplex DNA had certain feasibility.

**Figure 3:**
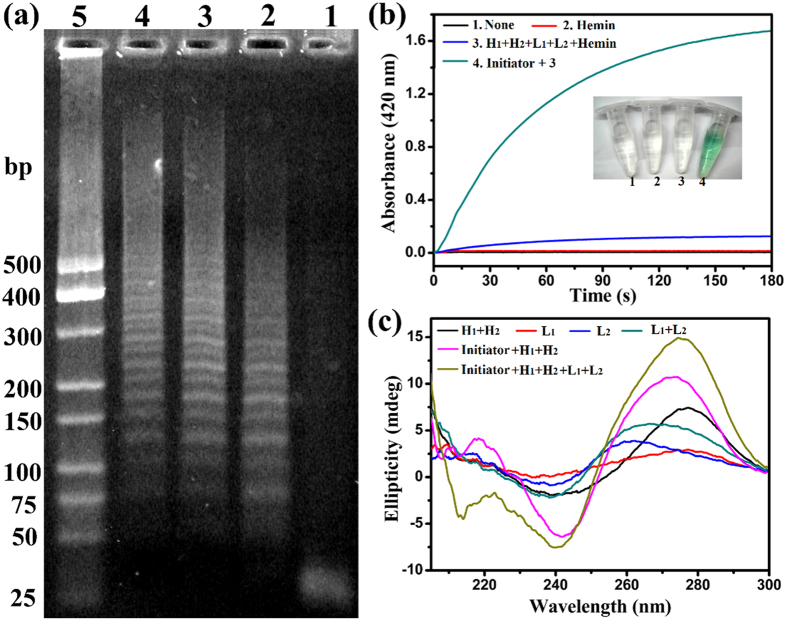
(**a**) Effect of Initiator concentrations on HCR. Lane 1-4, four different concentrations of Initiator (0.0 μM, 1.0 μM, 0.4 μM and 0.1 μM) in the mixture of 1.0 μM H_1_ and 1.0 μM H_2_; lane 5, 25-bp ladder marker. (**b**) The time-dependent absorbance changes of the H_2_O_2_-ABTS^2−^ reactions in different conditions and corresponding color changes. (10 nM Initiator, 0.2 μM H_1_, 0.2 μM H_2_, 0.4 μM L_1_, 0.4 μM L_2_, 0.4 μM hemin, 2.0 mM H_2_O_2_ and 0.2 mM ABTS^2−^) (**c**) Circular dichroism (CD) spectroscopy in different cases.(5 μM Initiator, 5 μM H_1_, 5 μM H_2_, 10 μM L_1_, 10 μM L_2_).

To ensure the success of HCR reaction as designed, the agarose gel electrophoresis assay was employed. As depicted in Fig. 3a, from lane 1, lane 2, lane 3 to lane 4, the concentrations of Initiator DNA were 0.0 μM, 1.0 μM, 0.4 μM, 0.1 μM, respectively. In the absence of Initiator, the HCR reaction could not carry out because there was only one band of H_1_ and H_2._ By the addition of different concentration Initiator, the HCR reactions took place, and the band of H_1_ and H_2_ disappeared. It was noted that the concentration of Initiator was lower, the length of HCR products was longer. It suggested that higher concentration of Initiator opened more H_1_, which led more parallel polymerization reactions to carry out at the same time, generating shorter HCR products, which was in good accordance with some previous literatures^20^,^21^,^22^. In order to verify the feasibility of the proposed method, the H_2_O_2_-ABTS^2−^ reactions were investigated under different conditions. (Figure 3b) The absorbance of the mixture of H_2_O_2_ and ABTS^2−^ had little change within 180 s (curve (1)). Similarly, by the addition of hemin (Fe-protoporphyrin IX), an iron-containing prosthetic group among a diverse group of proteins, with weak peroxidase-like activity, the absorbance of the mixture H_2_O_2_ and ABTS^2−^ also had inappreciable change within 180 s as well (curve (2))^23^,^24^. In the absence of Initiator, the absorbance of the mixture H_1_, H_2_, L_1_, L_2_, hemin, H_2_O_2_ and ABTS^2−^ had weak change within 180 s (curve (3)), due to the mildly enhanced peroxidase-like activity of hemin by abridged guanine-rich single-strand sequences L_2_ possessing three GGG repeats. Upon addition of Initiator, the absorbance of the mixture H_1_, H_2_, L_1_, L_2_, hemin, H_2_O_2_ and ABTS^2−^ occurred obvious change (curve (4)). These results suggested that Initiator disturbed the steady state of H_1_ and H_2_. The resulting HCR products were successfully recognized by L_1_ and L_2_. Through the synergistic effect, the triplex DNA and asymmetrically split G-quadruplex configurations were formed. The special G-quadruplex structure bound with hemin and formed the asymmetrically split G-quadruplex/hemin complex with high peroxidase-like activity, which could catalyze ABTS^2−^ and H_2_O_2_. The colored signals visible to the naked eye were in good accordance with that of the absorbance changes at 420 nm. Therefore, the proposed strategy was feasibility and it worked as designed.

Circular dichroism (CD) spectroscopy has great superiority in exploring the DNA configuration. To further understand the principle of the proposed assay, CD spectroscopy was measured under different cases. As displayed in Fig. 3c, there was no apparent characteristic peak for H_1_ + H_2_, L_1_, L_2_, L_1_ + L_2_, respectively. However, by the addition of Initiator, the CD spectroscopy of H_1_ + H_2_ had a positive cotton effect peak at about 218 nm, an enhanced positive peak at about 270 nm and negative peak at about 242 nm. It indicated that Initiator triggered the HCR between H_1_ and H_2_, and produced a chain-like assembly of H_1_ and H_2_. Moreover, by the introduction of L_1_ and L_2_, the CD spectroscopy of the mixture (Initiator + H_1_ + H_2_) had an obvious negative peak appeared at about 210 nm, which was characterized as the forming of triplex DNA^25^,^26^. There was a positive peak near 270 nm and negative band around 240 nm in the CD spectroscopy of Initiator + H_1_ + H_2_ + L_1_ + L_2_, suggesting that the typical parallel G-quadruplex structures were formed by L_1_ and L_2_ with the help of triplex forming^27^.

### Effect of the experimental conditions

**Figure 4:**
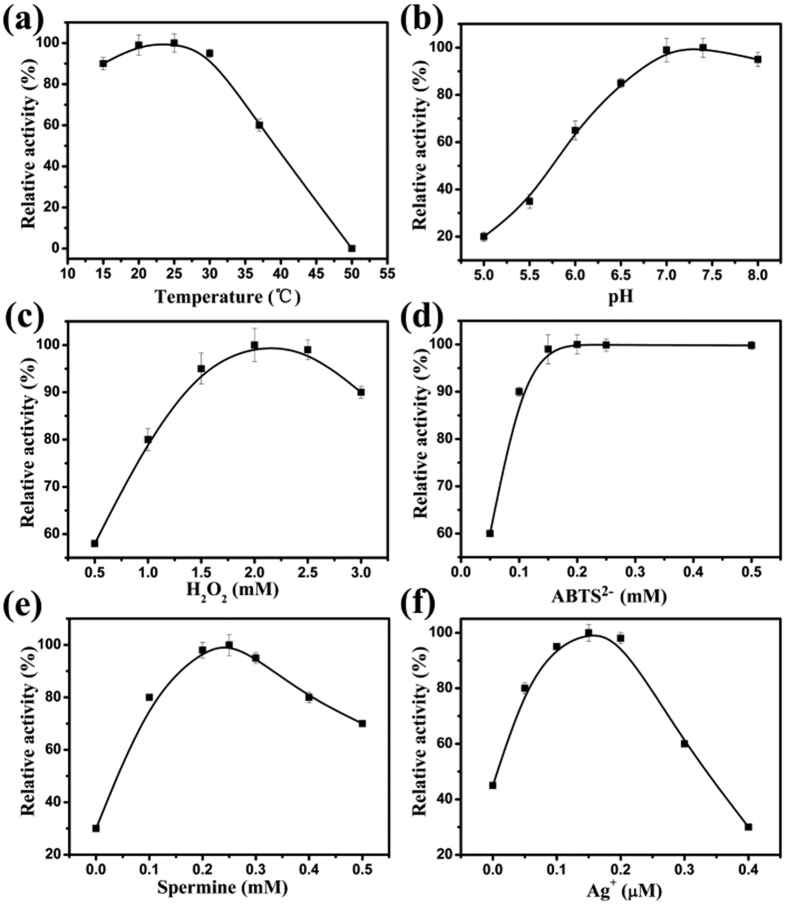
Effect of different conditions on the time-dependent absorbance changes of the H_2_O_2_-ABTS^2−^ reactions, involving the temperature of the reaction solution (**a**), pH value of Tris-Ac buffer (**b**), concentrations of H_2_ O_2_ (**c**), ABTS^2−^ (**d**), spermine (**e**) and Ag^+^ (**f**). The error bars represent the standard deviation of the three measurements. The optimal point in each curve was set as 100%.

The proposed colorimetric sensing system was established based upon recognizing and detecting the HCR product by means of synergy effect of triplex DNA and the asymmetrically split G-quadruplex forming, which bound with hemin and catalyzed the reaction of ABTS^2−^ and H_2_O_2_. Herein, some key factors included in the system may affect the sensitivity of the colorimetric assay, such as the temperature and pH of the reaction solution, concentrations of H_2_O_2_, ABTS^2−^, spermine and Ag^+^. Upon the optimization, temperature of the reaction solution, pH value of Tris-Ac buffer, the concentrations of H_2_O_2_, ABTS^2−^, spermine and Ag^+^ were 25 °C, pH 7.4, 2.0 mM, 0.20 mM, 0.25 mM, 0.15 μM, respectively (Fig. 4). The details were depicted in Supplementary Information.

### Sensitivity of the proposed method

**Figure 5:**
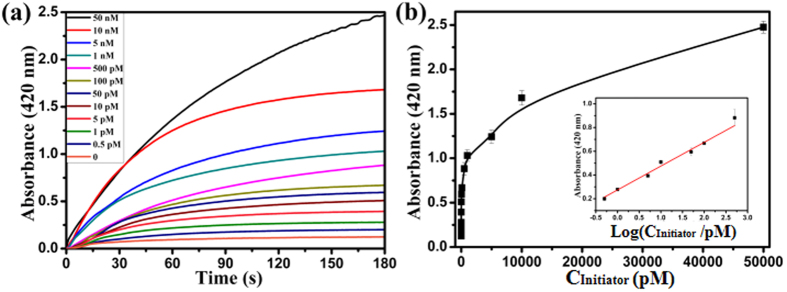
(**a**) The time-dependent absorbance changes of ABTS^·−^ corresponding to different concentrations of Initiator. (**b**) The calibration curve of the absorbance changes observed after a fixed time interval of 180 s *vs* varible concentrations of Initiator. Inset shows the linear relationship between the absorbance value of the oxygenation product ABTS^·−^ (λ_max_ = 420 nm) at the 180 seconds time point and the logarithm of Initiator concentrations. The error bars represent the standard deviation of the three measurements.

Under optimal experimental conditions, the sensitivity and dynamic range of the colorimetric HCR assay were evaluated toward Initiator standards in 20 mM Tris-Ac, pH 7.4, 25 °C containing 2.0 mM H_2_O_2_, 0.20 mM ABTS^2−^, 0.25 mM Spermine, 0.15 μM Ag^+^, 0.10 M K^+^, 0.40 μM hemin, 0.20 μM H_1_, 0.20 μM H_2_, 0.40 μM L_1_ and 0.40 μM L_2_. Figure 5a displayed the time-dependent absorbance changes of ABTS^·−^ upon different concentrations of Initiator. When the concentration of Initiator was higher, the absorbance changes increased in their intensities. In the absence of Initiator, feeble absorbance change was observed. Thus, the blank response of the time-dependent absorbance changes of ABTS^·−^ was negligible. The calibration curve corresponding to the absorbance changes observed after a fixed time interval of 180 s upon analysis of different concentrations of Initiator was described in Fig. 5b. The calibration plots (Fig. 5b, Inset) exhibited a linear relationship between the absorbance value of the oxygenation product ABTS^·−^ (λ_max_ = 420 nm) and the logarithm of Initiator concentrations in the range from 0.5 pM to 500 pM, the linear regression equation was A = 0.2094 Log (C_Initiator_/pM) + 0.2743, where A was the absorbance value of ABTS^·−^ (λ_max_ = 420 nm) at the 180 seconds time point, C_Initiator_ was the concentration of Initiator. The correlation coefficient R was 0.9937. The detection limit was 0.1 pM (3σ/slope). Compared to the previously reported DNA colorimetric sensing strategies (as shown in Supplementary Table S1)^13^,^17^,^28^,^29^, the present work possessed high sensitivity.

### Generality of the proposed method

**Figure 6:**
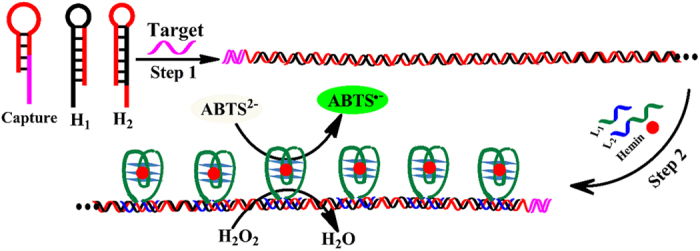
The generality of the proposed method for other nucleic acid sequences.

The proposed method is able to detect other nucleic acid sequences, including miRNA and DNA, with the aid of elaborately designed hairpin Capture probes (as shown in Fig. 6). The Capture probe consists of two main parts. One is the Target recognition element represented by pink color, which is in the stem and sticky end of the hairpin Capture probe, in order to easily recognized Target nucleic acid sequence. The other is the Signal amplification element expressed in red color, which mainly concentrates in the loop of the Capture hairpin structure, to avoid non-specially triggering HCR. In the absence of Target nucleic acid sequence, the Signal amplification DNA sequence is firmly locked in the hairpin Capture probe. Thus, no HCR happens and no color change is discovered. In the presence of Target nucleic acid sequence, the Capture hairpin structure is opened and the Signal amplification DNA sequence is released. Hence, HCR takes place and the characteristic green color is observed. These suggest the generality of the proposed method in nucleic acid detection. Furthermore, the proposed method can be extended to assay other targets, by using aptamer technology, such as small molecules, proteins and cells^30^,^31^,^32^.

### Circulating tumor DNA (ctDNA) detection

**Figure 7:**
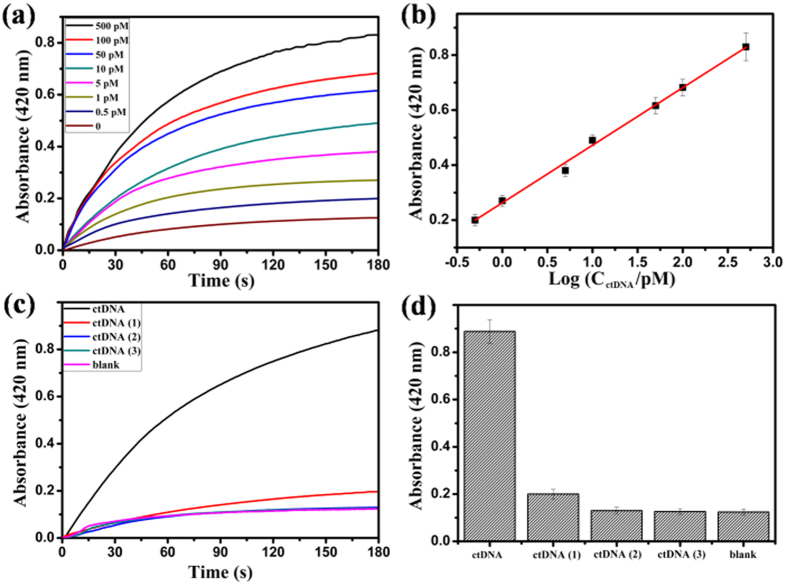
(**a**) The time-dependent absorbance changes of ABTS^·−^ corresponding to different concentrations of ctDNA. (**b**) The linear relationship between the absorbance value of the oxygenation product ABTS^·−^ (λ_max_ = 420 nm) at the 180 seconds time point and the logarithm of ctDNA concentrations. (**c**) Time-dependent absorbance changes upon analysis of different analytes: ctDNA, 500 pM; ctDNA (1), one-base mutant, 500 pM; ctDNA (2), two-base mutant, 500 pM; ctDNA (3), three-base mutant, 500 pM; no analyte. (**d**) The calibration column corresponding to the absorbance changes observed after a fixed time interval of 180 s. The error bars represent the standard deviation of the three measurements.

As a proof-of-concept, a circulating tumor DNA (ctDNA) was chosen as the model analyte of interest, which was a remarkable biomarker due to the high frequency of mutations of the PIK3CA gene in breast cancer (PIK3CA E542 KM (c.1624 G > A), 5′-AGT GAT TTT AGA GAG AG-3′)^33^. The hairpin DNA sequence (5′-AGA GAG AGA AGA GAG GAA GAG GAA CTC TT CT CTC TCT AAA ATC ACT-3′) was introduced as Capture probe to recognize ctDNA. In the presence of ctDNA, the Capture probe was opened and released a single-strand DNA sequence, which initiated the cross-opening of hairpins H_1_ and H_2_. The chain-like HCR products were characterized by the agarose gel electrophoresis assay. As depicted in Supplementary Figure S1, lane 1 only contained 1.0 μM Capture, lane 2 had 1.0 μM Capture and 1.0 μM ctDNA. In the presence of 1.0 μM Capture, 1.0 μM H_1_, and 1.0 μM H_2_, the concentrations of ctDNA from lane 3, lane 4, lane 5 to lane 6, were 0.0 μM, 1.0 μM, 0.4 μM, 0.1 μM, respectively. It suggested that by the addition of ctDNA, the Capture was opened and released a single-strand DNA sequence which initiated the HCR reaction. Figure 7a depicted the time-dependent absorbance changes of ABTS^·−^ upon analysis of different concentrations of ctDNA. As the concentration of ctDNA was lower, the absorbance changes weakened in their intensities. In the absence of ctDNA, the blank response of the time-dependent absorbance changes of ABTS^·−^ could be negligible. The calibration curve corresponding to the absorbance changes observed after a fixed time interval of 180 s upon analysis of different concentrations of ctDNA was described in Fig. 7b. The absorbance value of ABTS^·−^ (λ_max_ = 420 nm) at the 180 seconds time point showed a good linear correlation with the logarithm of ctDNA concentrations in the range from 0.5 pM to 500 pM, with a detection limit as low as 0.1 pM (3σ/slope) and the correlation coefficient R 0.9956. Compared to some other reported ctDNA detection methods (as shown in Supplementary Table S2)^33^,^34^,^35^, this approach has the following good performances: (1) With the aid of triple-helix DNA forming, the HCR amplification and the high peroxidase-like catalytic activity of the G-Quadruplex DNAzymes are combined, and thus, high sensitive colorimetric signals can be achieved for ctDNA detection. (2) The signatures of enzyme-free and visualization give this approach a potential for point-of-care testing in clinical analysis.

To validate the specificity of ctDNA detection system, three sequences of the one-, two-, and three-base mutations ctDNA (1), ctDNA (2), and ctDNA (3), respectively, were prepared as analytes to test. Figure 7c depicted the time-dependent absorbance changes of ABTS^·−^ upon analysis of ctDNA, ctDNA (1), ctDNA (2), ctDNA (3), all at a concentration of 500 pM, and without any analytes. The calibration column corresponding to the absorbance changes observed after a fixed time interval of 180 s was demonstrated in Fig. 7d. The spectral changes of the two-base and three-base mismatched analytes ctDNA (2) and ctDNA (3) were almost identical to the background signal of the system. However, the one-base mismatched ctDNA (1) was easily distinguished from ctDNA. It suggested that the system had high selectivity.

To evaluate the feasibility of the assay in real samples, human blood plasma samples voluntarily provided by healthy people were tested and the DNA extraction procedures from blood plasma were described in *Methods*. To detect ctDNA in human blood plasma samples, 1: 10 dilution of human blood plasma samples was analyzed alone or spiked with 10 pM, 50 pM and 100 pM ctDNA. As shown in Table 1, the obtained results corresponded with the same concentrations provided in Tris-Ac buffer. The relative standard deviations (RSDs) for the three samples were all below 6%, exhibiting the adequate accuracy and good reproducibility of the present sensing strategy. It suggested that this colorimetric sensing had good anti-interference performance in the detection of ctDNA in blood plasma samples. Hence, the approach had a great potential in the field of cancer early diagnosis and drug resistance in advanced cancers.

In conclusion, an ultrasensitive colorimetric sensing platform for circulating tumor DNA (ctDNA) was established, by using triple-helix DNA forming as a pivot to combine the hybridization chain reaction (HCR) amplification and the high peroxidase-like catalytic activity of the G-Quadruplex DNAzymes. This work provides a feasible method for the quantitative detection of ctDNA with high sensitivity, selectivity, and a detection limit of 0.1 pM (3σ/slope), as well as satisfactory accuracy and reproducibility in human blood plasma samples. This design strategy can be used not only for ctDNA detection, but also can be further expanded to other biomarkers, involving other nucleic acid sequences, small molecules, proteins and cells, based on the elaborate design of the structures of capture probes. Although this study offers a costless and simple sensing platform for colorimetric and visual detection of ctDNA with a highly sensitive signal and negligible background, it still does not meet the needs of the clinical analysis. Therefore, the later studies will focus on the sensitivity and point-of-care testing of the strategies for nucleic acid detection.

## Methods

### Materials

All oligonucleotides, 2,2′-azino-bis(3-ethylbenzothiozoline-6-sulfonic acid) (ABTS) and hemin (from bovine) were obtained from Sangon Biotech. Co. Ltd. (Shanghai, China). All reagents were of analytical grade and used as received without further purification. The stock solutions of all DNA molecules (100 μM) were prepared in 20 mM Tris-Ac buffer (pH 7.4) and accurately quantified using UV-vis absorption spectrum with the following extinction coefficients (260 nm, M^−1^cm^−1^): (A) 15400, (G) 11500, (C) 7400, (T) 8700. The stock solution of 4.0 mM hemin was prepared in dimethyl sulfoxide (DMSO), stored in darkness at −20 °C. All oligonucleotides solutions were diluted to required concentrations with the 20 mM Tris-Ac buffer (pH 7.4) and heated at 90 °C for 10 min and then cooled down naturally to room temperature prior to use. Nanopure water (18.1 MΩ) was used in this study produced by a 350 Nanopure water system (Guangzhou Crystalline Resource Desalination of Sea Water and Treatment Co. Ltd.). The sequences of all DNA molecules involved in this study were depicted as follows:

H_1_: 5′-ttc ctc TTC CTC TCT TCT CTC TCT tcc tct AGA GAG AGA AGA GAG GAA-3′

H_2_: 5′-AGA GAG AGA AGA GAG GAA gag gaa TTC CTC TCT TCT CTC TCT aga gga-3′

Initiator: 5′-AGA GAG AGA AGA GAG GAA GAG GAA-3′

L_1_: 5′-TGG GT TCT CTC CTT CTC CTT-3′

L_2_: 5′-TCT CCT TCT CTC TCT TGG GTA GGG CGG G-3′

dsDNA: 5′-TTC CTC TTC CTC TCT TCT CTC TCT TCC TCT-3′

3′-AAG GAG AAG GAG AGA AGA GAG AGA AGG AGA-5′

ctDNA: 5′-AGT GAT TTT AGA GAG AG-3′

ctDNA (1): 5′-AGT GAT TTA AGA GAG AG-3′

ctDNA (2): 5′-AGA GAT TTA AGA GAG AG-3′

ctDNA (3): 5′-AGA GAT TTA AGA GTG AG-3′

Capture: 5′-AGA GAG AGA AGA GAG GAA GAG GAA CTCTT CT CTC TCT AAA ATC ACT-3′

### Circular dichroism studies

Circular dichroism (CD) spectroscopy experiments were measured on the Jasco J-810-150S spectropolarimeter at room temperature employing a quartz cell with a 0.1 cm path length. CD spectra were collected from 205 to 300 nm and with a scanning speed of 100 nm/min and 0.2 nm intervals. All CD spectra were baseline-corrected for signal contributions due to the buffer and were the average of at least three runs. The process was as follows: Samples H_1_ + H_2_, L_1_, L_2_, L_1_ + L_2_, Initiator + H_1_ + H_2_, Initiator + H_1_ + H_2_ + L_1_ + L_2_, prepared respectively in the Tris-Ac buffer (pH 7.4) containing 20 mM Tris, 0.25 mM spermine, 0.15 μM Ag^+^, 0.10 M K^+^, were first incubated at 37 °C for 3 h, and then transfered to 25 °C water bath environment and hatched for 1 h prior to the CD measurement. The final concentrations of Initiator, H_1_, H_2_, L_1_, L_2_, involved in the CD studies were 5 μM, 5 μM, 5 μM, 10 μM, 10 μM, respectively.

### Agarose gel electrophoresis

The 2% agarose gel contained 1 × 4 S Red Plus Nucleic Acid Stain, freshly prepared at laboratory, was used to perform the agarose gel electrophoresis analysis. The loading sample was mixed with 10 μL of DNA sample, 2 μL 6 loading buffer. The electrophoresis was carried out at a constant voltage of 100 V for 90 min, then the gel was imaged with a digital camera under UV irradiation.

### Native polyacrylamide gel electrophoresis (PAGE)

Nondenaturing polyacrylamide gel (15%) was prepared in 50 mM, pH 6.0 Tris-Ac buffer. Before loading samples, 10 μL oligonucleotides was mixed with 2 μL of 6 × loading buffer. Gel electrophoresis was run at 25 °C for 2 h under a voltage of 90 V/cm at pH 6.0. After staining with 4 S Red Plus Nucleic Acid Stain for 10 min and finally photographed with a digital camera under the illumination of UV light.

### Initiator assay procedure

Samples contained different concentration Initiator, 0.2 μM H_1_, 0.2 μM H_2_, 0.4 μM L_1_, 0.4 μM L_2_, prepared in the Tris-Ac buffer (pH 7.4) containing 20 mM Tris, 0.25 mM spermine, 0.15 μM Ag^+^, 0.10 M K^+^, 0.4 μM hemin, were first incubated at 37 °C for 3 h, and then transfered to 25 °C water bath environment and hatched for 1 h. Finally, ABTS and H_2_O_2_ were added, the final concentrations of each were 0.2 mM, 2.0 mM, respectively. The detection was carried out in the Kinetics mode of a UV-2550 UV-vis spectrophotometer (Shimadzu, Japan). The maximum absorption wavelength of the radical anion ABTS^·−^ (the oxidation product of ABTS^2−^) was about 420 nm.

### ctDNA assay procedure

Samples contained different concentration ctDNA, 0.2 μM Capture, 0.2 μM H_1_, 0.2 μM H_2_, 0.4 μM L_1_, 0.4 μM L_2_, prepared in the Tris-Ac buffer (pH 7.4) containing 20 mM Tris, 0.25 mM spermine, 0.15 μM Ag^+^, 0.10 M K^+^, 0.4 μM hemin, were first incubated at 37 °C for 3 h, and then transfered to 25 °C water bath environment and hatched for 1 h. Finally, ABTS and H_2_O_2_ were added, the final concentrations of each were 0.2 mM, 2.0 mM, respectively. The detection was carried out in time course mode by monitoring the absorbance change at 420 nm on a UV-2550 UV-vis spectrophotometer (Shimadzu, Japan).

### Sample preparation

The study protocol was approved by the ethic committee of Zhongshan Medical College, Sun Yat-sen University. Informed consent was obtained from each subject. All these experiments were performed in accordance with the relevant guidelines and regulations. Blood samples were gathered from healthy volunteers in our lab. Plasma samples were obtained by centrifugation of blood for about 5 min with a rotation rate of 3000–4000 rpm.

### Samples analysis

DNA was isolated using the EZ-10 Spin Column Blood Genomic DNA Purification Kit (Sangon Biotech.) following the manufacturer’s protocol. The procedure is depicted simply as follows: First, 100 μL of PBS solution was added in 100 μL plasma sample, and the solution was mixed. And then the aboved solution mixed with 20 μL Proteinase K, 200 μL Buffer CL was added, the obtained solution was stirred vigorously for well mixing, incubating for 10 min at 56 °C water bath. Second, 200 μL ethyl alcohol was added to the above solution, and stirred vigorously for complete mixing. Transfer the prepared mixture to the spin column. Centrifuge for 1 min at 8,000 rpm. Discard the collection tube containing the flow-through solution. Place the column into a new 2 mL collection tube. Add 500 μL of CW1 solution (with ethanol added). Centrifuge for 1 min at 10,000 rpm. Discard the flow-through and place the column back into the collection tube. Add 500 μL of CW2 solution (with ethanol added) to the column. Centrifuge for 2 min at maximum speed ( ≥ 12,000 rpm). Discard the collection tube containing the flow-through solution and transfer the column to a sterile 1.5 mL microcentrifuge tube (not included). Add 50 μL of CE Buffer to the center of the column membrane to elute DNA. Incubate for 3 min at room temperature and centrifuge for 2 min at 12,000 rpm. Discard the purification column. Use the purified DNA immediately in downstream applications. The following DNA analysis is similar to the ctDNA assay procedure.

## Additional Information

**How to cite this article:** Li, R. *et al*. Ultrasensitive colorimetric detection of circulating tumor DNA using hybridization chain reaction and the pivot of triplex DNA. *Sci. Rep.* **7**, 44212; doi: 10.1038/srep44212 (2017).

**Publisher's note:** Springer Nature remains neutral with regard to jurisdictional claims in published maps and institutional affiliations.

## Supplementary Material

Supplementary Information

## Figures and Tables

**Table 1:** The proposed method for the detection of ctDNA in human blood plasma samples.

ctDNA (pM)				
Sample	Added	Found Mean^a^ ± SD^b^	Recovery (%)	RSD^c^ (%)
1	0	—	—	—
2	10	9.3 ± 0.5	98	5.38
3	50	44.5 ± 2.5	94	5.62
4	100	95.6 ± 5.4	101	5.65

^a^The mean of three determinations. ^b^SD represents standard deviation. ^c^RSD repersents relative standard deviation.

## References

[b1] Bidard F., Weigelt B. & Reis-Filho J. Going with the Flow: From Circulating Tumor Cells to DNA. Sci. Transl. Med. 5, 207ps14 (2013). 10.1126/scitranslmed.3006305 24132635

[b2] Newman A. . An ultrasensitive method for quantitating circulating tumor DNA with broad patient coverage. Nat. Med. 20, 548–554 (2014). 24705333 10.1038/nm.3519 PMC4016134

[b3] Das J., Ivanov I., Sargent E. H. & Kelley S. DNA Clutch Probes for Circulating Tumor DNA Analysis. J. Am. Chem. Soc. 138, 11009–11016 (2016). 27513828 10.1021/jacs.6b05679

[b4] Jr L. & Bardelli A. Liquid Biopsies: Genotyping Circulating Tumor DNA. J. Clin. Oncol. 32, 579–586 (2014). 24449238 10.1200/JCO.2012.45.2011 PMC4820760

[b5] Monsur A. . Rolling circle amplification: a versatile tool for chemical biology, materials science and medicine. Chem. Soc. Rev. 43, 3324–3341 (2014). 24643375 10.1039/c3cs60439j

[b6] Zhang J. . Hairpin DNA-templated silver nanoclusters as novel beacons in strand displacement amplification for microRNA detection. Anal. Chem. 88, 1294–1302 (2016). 26675240 10.1021/acs.analchem.5b03729

[b7] Garcia E., Farnleitner A., Mach R., Krska R. & Brunner K. A rapid genomic DNA extraction method and its combination with helicase dependent amplification for the detection of genetically modified maize. Anal. Methods, 8, 136–141 (2016).

[b8] Dirks R. & Pierce N. Triggered amplification by hybridization chain reaction. Proc. Natl. Acad. Sci. USA 101, 15275–15278 (2004). 15492210 10.1073/pnas.0407024101 PMC524468

[b9] Choi H., Beck V. & Pierce N. Next-Generation *in Situ* Hybridization Chain Reaction: Higher Gain, Lower Cost, Greater Durability. ACS Nano 8, 4284–4294 (2014). 24712299 10.1021/nn405717p PMC4046802

[b10] Ikbal J., Lim G. & Gao Z. The hybridization chain reaction in the development of ultrasensitive nucleic acid assays. Trends Anal. Chem. 64, 86–99 (2015).

[b11] Li X., Wang Y., Wang L. & Wei Q. A surface plasmon resonance assay coupled with a hybridization chain reaction for amplified detection of DNA and small molecules. Chem. Commun. 50, 5049–5052 (2014). 10.1039/c4cc01374c 24714922

[b12] Ding X. . An enzyme-free surface plasmon resonance biosensing strategy for detection of DNA and small molecule based on nonlinear hybridization chain reaction. Biosens. Bioelectron. 87, 345–351 (2017). 27587359 10.1016/j.bios.2016.08.077

[b13] Liu P. . Enzyme-Free Colorimetric Detection of DNA by Using Gold Nanoparticles and Hybridization Chain Reaction Amplification. Anal. Chem. 85, 7689–7695 (2013). 23895103 10.1021/ac4001157

[b14] Miao P., Tang Y. & Yin J. MicroRNA detection based on analyte triggered nanoparticle localization on a tetrahedral DNA modified electrode followed by hybridization chain reaction dual amplification. Chem. Commun. 51, 15629–15632 (2015). 10.1039/c5cc05499k 26376704

[b15] Miao J. . A plasmonic colorimetric strategy for visual miRNA detection based on hybridization chain reaction. Sci Rep. 6, 32219 (2016). 27534372 10.1038/srep32219 PMC4989231

[b16] Xie P. . Highly sensitive detection of lipopolysaccharides using an aptasensor based on hybridization chain reaction. Sci Rep. 6, 29524 (2016). 27404735 10.1038/srep29524 PMC4941573

[b17] Zou . Colorimetric sensing platform based upon recognizing hybridization chain reaction product with oligonucleotide modified gold nanoparticles through triplex formation Nanoscale 9, 1986–1992 (2017). 10.1039/c6nr09089c 28106202

[b18] Deng M., Zhang D., Zhou Y. & Zhou X. Highly Effective Colorimetric and Visual Detection of Nucleic Acids Using an Asymmetrically Split Peroxidase DNAzyme. J. Am. Chem. Soc. 130, 13095–13102 (2008). 18763776 10.1021/ja803507d

[b19] Ihara T., Ishii T., Araki N., Wilson A. & Jyo A. Silver Ion Unusually Stabilizes the Structure of a Parallel-Motif DNA Triplex. J. Am. Chem. Soc. 131, 3826–3827 (2009). 19243184 10.1021/ja809702n

[b20] Ge Z. . Hybridization Chain Reaction Amplification of MicroRNA Detection with a Tetrahedral DNA Nanostructure-Based Electrochemical Biosensor. Anal. Chem. 86, 2124–2130 (2014). 24495151 10.1021/ac4037262

[b21] Niu S., Jiang Y. & Zhang S. Fluorescence detection for DNA using hybridization chain reaction with enzyme-amplification. Chem. Commun. 46, 3089–3091 (2010). 10.1039/c000166j 20424746

[b22] Chen Y. . *In Situ* Hybridization Chain Reaction Amplification for Universal and Highly Sensitive Electrochemiluminescent Detection of DNA. Anal. Chem. 84, 7750–7755 (2012). 22924989 10.1021/ac3012285

[b23] Li R. . G-quadruplex DNAzymes-induced highly selective and sensitive colorimetric sensing of free heme in rat brain. Analyst 139, 1993–1999 (2014). 24600682 10.1039/c3an02025h

[b24] Li R., Xiong C., Xiao Z. & Ling L. Colorimetric detection of cholesterol with G-quadruplex-based DNAzymes and ABTS^2−^. Anal. Chim. Acta 724, 80–85 (2012). 22483213 10.1016/j.aca.2012.02.015

[b25] Manzini G., Xodo L. & Gasparotto D. Triple Helix Formation by Oligopurine-oligopyrimidine DNA Fragments. J. Mol. Biol. 213, 833–843 (1990). 2359124 10.1016/S0022-2836(05)80267-0

[b26] Feng L., Huang Z., Ren J. & Qu X. Toward site-specific, homogeneous and highly stable fluorescent silver nanoclusters fabrication on triplex DNA scaffolds. Nucl. Acids Res 40, e122 (2012). 22570417 10.1093/nar/gks387 PMC3439878

[b27] Nakayama S. & Sintim H. Colorimetric Split G-Quadruplex Probes for Nucleic Acid Sensing: Improving Reconstituted DNAzyme’s Catalytic Efficiency via Probe Remodeling. J. Am. Chem. Soc. 131, 10320–10333 (2009). 19621970 10.1021/ja902951b

[b28] Dong J., Cui X., Deng Y. & Tang Z. Amplified detection of nucleic acid by G-quadruplex based hybridization chain reaction. Biosens. Bioelectron. 38, 258–263 (2012). 22739472 10.1016/j.bios.2012.05.042

[b29] He H. . Unusual sequence length-dependent gold nanoparticles aggregation of the ssDNA sticky end and its application for enzyme-free and signal amplified colorimetric DNA detection. Sci. Rep. 6, 30878 (2016). 27477392 10.1038/srep30878 PMC4967886

[b30] Stoltenburg R., Krafčiková P., Víglaský V. & Strehlitz B. G-quadruplex aptamer targeting Protein A and its capability to detect Staphylococcus aureus demonstrated by ELONA. Sci. Rep. 6, 33812 (2016). 27650576 10.1038/srep33812 PMC5030626

[b31] Yang Z., Castrignanò E., Estrela P., Frost C. G. & Kasprzyk-Hordern B. Community Sewage Sensors towards Evaluation of Drug Use Trends: Detection of Cocaine in Wastewater with DNA-Directed Immobilization Aptamer Sensors. Sci. Rep. 6, 21024 (2016). 26876971 10.1038/srep21024 PMC4753446

[b32] Chang Y. . Rapid single cell detection of Staphylococcus aureus by aptamer-conjugated gold nanoparticles. Sci. Rep. 3, 1863 (2013). 23689505 10.1038/srep01863 PMC3659324

[b33] Zhou Q. . Detection of Circulating Tumor DNA in Human Blood via DNA Mediated Surface-Enhanced Raman Spectroscopy of Single-Walled Carbon Nanotubes. Anal. Chem. 88, 4759–4765 (2016). 27028517 10.1021/acs.analchem.6b00108

[b34] Nguyen A. H. & Sim S. J. Nanoplasmonic biosensor: Detection and amplification of dual bio-signatures of circulating tumor DNA. Biosens. Bioelectron. 67, 443–449 (2015). 25220802 10.1016/j.bios.2014.09.003

[b35] Das J. . An electrochemical clamp assay for direct, rapid analysis of circulating nucleic acids in serum. Nat. Chem. 7, 569–575 (2015). 26100805 10.1038/nchem.2270

