# Supplementary material for: Ultrasensitive colorimetric detection of circulating tumor DNA using hybridization chain reaction and the pivot of triplex DNA
Source: Sci Rep. 2017 Mar 9;7:44212. doi: 10.1038/srep44212 (PMC5343571; doi:10.1038/srep44212)
Supplement: Supplementary Information [file srep44212-s1.pdf]

Supplementary Information

Ultrasensitive colorimetric detection of circulating tumor DNA using hybridization chain reaction and the pivot of triplex DNA

Ruimin Li, ‡ Li Zou, ‡ Yanwei Luo, Manjun Zhang, and Liansheng Ling*

School of Chemistry, Sun Yat-Sen University, Guangzhou 510275, P. R. China

‡Ruimin Li and Li Zou contributed equally to this work.

* Correspondence and requests for materials should be addressed to L.L. (email: cesllsh@mail.sysu.edu.cn)

Effect of the experimental conditions. The temperature of the reaction solution and pH value of Tris-Ac buffer are important to the catalytic activity of the asymmetrically split G-quadruplex/hemin DNAzymes. For the purpose of getting high sensitivity, temperature of the reaction solution and pH value of Tris-Ac buffer in the proposed colorimetric sensing system were investigated over the range of 15-50 °C and pH 5.0-8.0, respectively. As demonstrated in Figure 4a, the asymmetrically split G-quadruplex/hemin DNAzymes had high catalytic activity toward the reaction of $ABTS^{2-}$ and H_2O_2 in the range of 15-30 °C. However, the relative activity of the catalytic system declined rapidly at temperature above 37 °C. It was inferred that the high temperature had adverse impact on the forming of triplex DNA and the catalytic activity of the asymmetrically split G-quadruplex/hemin DNAzymes.[1,2] Thus, 25 °C was chosen as the optimal reaction temperature for the colorimetric assay. In the type of parallel triplex DNA, the composed C-G·C triads had well stability under acidic pH environment and could not be formed under physiological conditions. Recently, it was studied that Ag^+ had the ability to stabilize the forming of C-G·C triads under neutral pH environment.[3] The pH-dependent response curve of Tris-Ac buffer was shown in Figure 4b. The relative activity of the reaction mixture (2.0 mM H_2O_2, 0.2 mM $ABTS^{2-}$, 0.25 mM Spermine, 0.15 μM Ag^+, 25 °C) increased with the pH value increment of Tris-Ac buffer over the range of pH 5.0-7.0, and kept high activity over the range of pH 7.0-8.0. The phenomenon suggested that the triplex DNA was stable under neutral pH environment

with the aid of Ag^+ and the asymmetrically split G-quadruplex/hemin DNAzymes had high catalytic activity under neutral pH environment.[4] Therefore, pH 7.4 of Tris-Ac buffer was selected as the optimal pH reaction environment.

H_2O_2 and $ABTS^{2-}$ are essential to the color catalytic reaction system. Thus, to optimize the concentrations of H_2O_2 and $ABTS^{2-}$ is of importance for the sensitivity of the colorimetric assay. As shown in Figure 4c, the concentrations of H_2O_2 were investigated over the range of 0.50-3.0 mM. With the increase of H_2O_2, the relative activity of the reaction system increased in the range of 0.50-2.0 mM, and then weakened with the addition of H_2O_2. High concentration of H_2O_2 had negative impact on the catalytic activity of the asymmetrically split G-quadruplex/hemin DNAzymes. Herein, the optimized concentration of H_2O_2 was 2.0 mM in the color catalytic reaction system. The $ABTS^{2-}$-dependent response curve was depicted in Figure 4d. In the range of $ABTS^{2-}$ 0.05-0.15 mM, the relative activity of the colorimetric system increased with the augment of $ABTS^{2-}$ concentration, and kept unchangeable with the continued addition of $ABTS^{2-}$. Hence, the $ABTS^{2-}$ concentration 0.20 mM was used as optimal condition in the colorimetric system.

Spermine, a polycationic, low-molecular-weight molecule, not only has the ability to promote and stabilize triplex DNA structures, but also plays an important role in the activity enhancement of G-quadruplex/hemin DNAzyme.[5,6] Thus, the optimization of spermine concentration is necessary to ensure the high sensitivity of the color system. As shown in Figure 4e, the concentrations of spermine were investigated over the range of 0-0.50 mM. With the augment of spermine, the relative activity of the reaction system increased in the range of 0-0.25 mM, and then decreased with the addition of spermine. High concentration of spermine may cause the change of the structures of triplex DNA and the asymmetrically split G-quadruplex, and slackened the catalytic activity of the asymmetrically split G-quadruplex/hemin DNAzymes.[7] Herein, the optimized concentration of spermine was 0.25 mM in the color catalytic system. Since the G-quadruplex/hemin DNAzymes had high catalytic activity under neutral pH environment, construction of the stable triplex DNA under neutral pH was inevitable to the high sensitivity of the colorimetric assay. Ag^+ was known to stabilize the structure

of a parallel DNA triplex under neutral pH condition.[3] For the high sensitivity of the assay, the Ag^+ concentrations were studied over the range of 0-0.40 μM. As described in Figure 4f, by the introduction of Ag^+, the relative activity of the reaction system increased with the increase of Ag^+ concentration in the range 0-0.15 μM, but declined when Ag^+ concentration was higher than 0.20 μM. It was suggested that excess Ag^+ could chelate guanine bases at the binding sites involved in G-quadruplex formation, may disrupt these structures and inhibit the peroxidase activity of the asymmetrically split G-quadruplex/hemin.[8] Hence, the optimized concentration of Ag^+ was 0.15 μM in the color catalytic system.

References

(1) Li, R., Xiong, C., Xiao, Z. & Ling, L. Colorimetric detection of cholesterol with G-quadruplex-based DNAzymes and $ABTS^{2-}$. *Anal. Chim. Acta* **724**, 80-85 (2012).

(2) Yan, H., Xiong, C., Yuan, H., Zeng, Z. & Ling, L. Spacer Control the Dynamic of Triplex Formation between Oligonucleotide-Modified Gold Nanoparticles. *J. Phys. Chem. C* **113**, 17326-17331 (2009).

(3) Ihara, T., Ishii, T., Araki, N., Wilson, A. & Jyo, A. Silver Ion Unusually Stabilizes the Structure of a Parallel-Motif DNA Triplex. *J. Am. Chem. Soc.* **131**, 3826-3827 (2009).

(4) Xiao, Z., Guo, X. & Ling, L. Sequence-specific recognition of double-stranded DNA with molecular beacon with the aid of Ag^+ under neutral pH environment. *Chem. Commun.* **49**, 3573-3575 (2013).

(5) Beck, A., Vijayanathan, V., Thomas, T. & Thomas, T. J. Ionic microenvironmental effects on triplex DNA stabilization: Cationic counterion effects on poly(dT)·poly(dA)·poly(dT). *Biochimie* **95**, 1310-1318 (2013).

(6) Qi, C. et al. Activity enhancement of G-quadruplex/hemin DNAzyme by spermine. *RSC Adv.* **4**, 1441-1448 (2014).

(7) Musso, M. et al. Effects of Chain Length Modification and Bis(ethyl) Substitution of Spermine Analogs on Purine-Purine-Pyrimidine Triplex DNA Stabilization, Aggregation, and Conformational Transitions. *Biochemistry* **36**, 1441-1449 (1997).

(8) Zhou, X., Kong, D. & Shen, H. Ag^+ and Cysteine Quantitation Based on G-Quadruplex-Hemin DNAzymes Disruption by Ag^+. *Anal. Chem.* **82**, 789-793 (2010).

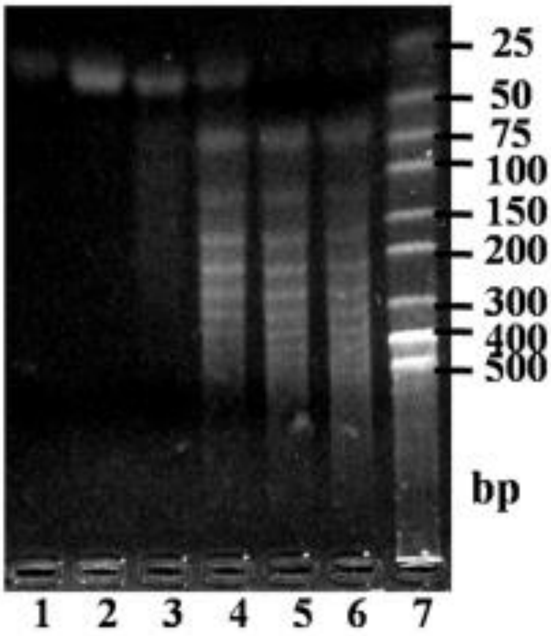

Figure S1. The agarose gel electrophoresis assay of HCR products.

Table S1. Comparison of colorimetric methods for DNA detection

Strategy	Linear range	Detection limit	Ref
HCR amplification & the asymmetrically split G-quadruplex/hemin	0.5-500 pM	0.1 pM	This work
HCR amplification & G-quadruplex/hemin	------	7.5 nM	28
HCR amplification & label-free AuNPs	0-0.5 nM	50 pM	29
catalytic hairpin assembly amplification & label-free AuNPs	50-300 pM	15 pM	30
HCR amplification & labeled AuNPs	10-500 pM	5 pM	17

Table S2. Comparison of the strategies for ctDNA detection

Detection method	Strategy	Linear range	Detection limit	Ref
Colorimetric	HCR amplification & the asymmetrically split G-quadruplex/hemin	0.5-500 pM	0.1 pM	This work
SERS	SWNTs & CuNP	10 fM -1 nM	1.5 fM	34
Refractometric	Peptide nucleic acids & immunogold colloids	------	0.05 pM	35
Electrochemical	Clamp	------	1 fg μl^{-1}	36
